# The combined diagnostic and therapeutic value of ultrasound and X-ray bone age index in girls with idiopathic central precocious puberty

**DOI:** 10.1530/EC-25-0354

**Published:** 2025-10-13

**Authors:** Linli Kan, Deng He, Wensheng Yue

**Affiliations:** ^1^ Department of Ultrasound Medicine, The Affiliated Hospital of North Sichuan Medical College, Sichuan Provincial Key Laboratory of Medical Imaging, Nanchong Municipal Key Laboratory of Ultrasonic Medical Engineering, Nanchong, China; ^2^ Department of Ultrasound Medicine, Suining Central Hospital, Suining, China; ^3^ Department of Radiology, Suining Central Hospital Radiology, Suining, China

**Keywords:** LASSO-logistic regression, idiopathic central precocious puberty (ICPP), ultrasound, bone age index (BAI), combined diagnosis, therapeutic efficacy evaluation

## Abstract

**Objectives:**

The objectives of this study are threefold: first, to evaluate the diagnostic utility of ultrasound in combination with radiographic bone age assessments for identifying idiopathic central precocious puberty (ICPP) in girls; second, to determine the efficacy of treatment; and third, to establish comprehensive models for both diagnosis and therapeutic evaluation.

**Methods:**

Female patients diagnosed with 96 cases of ICPP in our hospital from January 2022 to February 2024 were assigned to the research group, while 94 girls with premature thelarche from the same period were designated as the control group. Both groups underwent ultrasound examinations (of the uterus, ovaries, and breasts) and X-ray bone age evaluations, and their serum endocrine hormone levels (luteinizing hormone and follicle-stimulating hormone) were measured. Differences in ultrasound parameters, bone age indices, and hormone levels were analyzed between the research and control groups. Univariate and multivariate LASSO regression were employed to screen imaging parameters, and a LASSO-logistic regression model was established to create a combined predictive model. Receiver operating characteristic curves were plotted to investigate the diagnostic efficacy of the individual and combined models in ICPP. The general clinical data and imaging parameters of the ICPP group were compared before and after treatment, with ultrasound and bone age indices used in combination to assess therapeutic efficacy.

**Results:**

The LASSO regression screened important predictive indicators, including the bone age index (BAI), mean bilateral breast thickness, uterine longitudinal diameter, uterine anteroposterior diameter, uterine transverse diameter, endometrial thickness, mean bilateral ovarian length, and the mean number of ovarian follicles with a diameter >4 mm. These imaging parameters were incorporated into a logistic regression model, which demonstrated good discriminatory power with an AUC value of 0.895 (95% CI: 0.851, 0.938). The combined model outperformed the model using ultrasound alone (AUC: 0.869 (95% CI: 0.820, 0.918)) and the BAI model (AUC: 0.758 (95% CI: 0.690, 0.826)), showing superior discrimination and calibration. In the follow-up evaluation of the ICPP group post-treatment, ultrasound parameters such as uterine anteroposterior diameter, mean bilateral ovarian length, mean number of ovarian follicles with a diameter >4 mm, mean maximum follicular diameter, and mean bilateral ovarian volume showed good monitoring efficacy (AUC >0.7). In addition, the uterine longitudinal diameter and BAI exhibited high specificity. Together, these indicators achieved a combined diagnostic AUC value of 0.877 with 75% sensitivity and 87% specificity.

**Conclusions:**

To enhance diagnostic precision and overcome the constraints of single-metric evaluation approaches, a composite model was constructed by integrating ultrasound-based parameters with radiographic bone age measurements.

## Introduction

Precocious puberty (PP) is characterized by the onset of pubertal milestones before the typical age range for normal puberty. Specifically, it involves the development of secondary sexual characteristics in girls before the age of 8 and in boys before the age of 9, or the onset of menarche in girls before the age of 10 (1, 2). The incidence of PP is increasing annually due to the intricate interplay of lifestyle and sociocultural factors. Precocious puberty is categorized based on the activation status of the hypothalamic–pituitary–gonadal axis (HPGA) into three types: central precocious puberty (CPP), with idiopathic central precocious puberty (ICPP) being the most common; peripheral precocious puberty (PPP); and incomplete precocious puberty (IPP), where premature thelarche (PT) is the most prevalent form (3). Among these, ICPP and PT are the most prevalent. ICPP is typically characterized by accelerated growth velocity and advanced bone maturation, which may ultimately result in reduced adult height and psychological stress (4). According to Andrea *et al.* (5), adolescent girls with a history of ICPP are also at higher risk for polycystic ovarian syndrome (PCOS), underscoring the need for early diagnosis and timely intervention. In contrast, PT does not involve growth acceleration or advanced skeletal development and usually requires no treatment. The gonadotropin-releasing hormone (GnRH) stimulation test is regarded as the diagnostic gold standard for distinguishing ICPP from other subtypes, but its use is limited by low sensitivity, high cost, and the need for multiple blood draws (2, 6). Bone age (BA), reflecting skeletal maturity, serves as an objective and widely utilized marker in the assessment of precocious puberty. Traditional methods such as the Greulich-Pyle (GP) atlas and Tanner-Whitehouse (TW) scoring system are limited by their reliance on subjective interpretation. In recent years, AI-based BA assessment systems employing deep learning techniques have emerged, though most are still in developmental phases and lack standardized evaluation frameworks (7, 8). Compared with chronological age (CA) and traditional BA, the bone age index (BAI) demonstrates enhanced accuracy and lower variability, offering a more precise representation of developmental progress in children and adolescents (9, 19). Ultrasound examinations have been widely adopted in pediatric clinical evaluations due to their convenience, cost-effectiveness, and safety. Guidelines from various countries recommend performing a pelvic ultrasound as part of precocious puberty screening to identify potential causes and evaluate the internal reproductive organs, although specific cutoff values have not been established. Only a few national guidelines provide thresholds for uterine size, endometrial thickness, and ovarian measurements; however, these thresholds differ among countries (10). For example, China’s most recent guidelines suggest that pelvic ultrasound findings indicating a uterine length of 3.4–4.0 cm, ovarian volume of 1–3 mL, and the presence of multiple follicles ≥4 mm in diameter suggest the onset of puberty in girls (2). Current research on the combined use of ultrasound and radiography for diagnosis is limited both domestically and internationally, with inconsistent findings and few studies that have developed diagnostic models. Distinguishing between early-stage ICPP and isolated PT presents significant challenges. This study aims to explore the clinical value of combining ultrasound examinations with radiographic BA assessment to improve the diagnosis and treatment efficacy evaluation of ICPP in girls.

## Materials and methods

### Study population

All 96 girls diagnosed with ICPP at our clinic from January 2022 to February 2024 were enrolled as the case group, with a mean age of 7.73 ± 0.78 years. We also enrolled 94 girls with PT as the control group from the same period, with a mean age of 7.54 ± 0.75 years. The inclusion criteria for ICPP were as follows: girls under 8 years of age with breast development or under 10 years of age with menarche, and a positive GnRH stimulation test (peak luteinizing hormone (LH) ≥5.0 U/L and a ratio of peak LH to peak of follicle-stimulating hormone (FSH) ≥0.6). Inclusion criteria for PT: girls under 8 years old with isolated breast development and no other secondary sexual characteristics, with a negative GnRH stimulation test. Exclusion criteria: secondary true precocious puberty; comorbidities such as congenital adrenal hyperplasia, McCune-Albright syndrome, central nervous system abnormalities, primary hypothyroidism, or genes related to sexual development; non-compliance with follow-up or treatment. Before this study, the Hospital Ethics Committee (Approval No.: KYLLKS20240204) approved this study. Written consent was obtained from each participant or their guardians, and patient data were kept strictly anonymous and protected.

### Instruments and methods

Height was measured using the SZG-180 Children’s Height and Sitting Height Meter (Nantong Zilang Instrument & Equipment Co., Ltd, China), while weight was assessed with the TCS-200-RT electronic weight scale (Wuxi Weighing Apparatus Factory Co., Ltd, China). BA scans were conducted using a United Imaging uDR780i, and pituitary MRI images were obtained with a Signa Premier 3.0T superconducting MRI scanner. Ultrasound scans were performed using GE ViViD E95 and GE LOGIQ E9 color Doppler ultrasound diagnostic systems, featuring a 2–9 MHz convex array probe frequency and an 8–18 MHz linear probe frequency.

### General clinical data

Height and weight measurements were taken for all participants by the same pediatric nurse using identical anthropometric tools at the same time of day (08:00–10:00 h). Height was recorded in centimeters (cm) and weight in kilograms (kg), with both measurements rounded to one decimal place. Subsequently, the standard deviation scores (SDS) for height, weight, and BMI were computed for both the ICPP and PT groups. The formulas used were: target height (TH) = (father’s height + mother’s height − 13)/2; SDS = (X − mean)/SD; ΔSDS = measured height SDS − TH-SDS. Here, X represents the child’s measured current height, weight, and BMI; mean denotes the mean values for height, weight, and BMI of healthy girls of the same age; and SD is the standard deviation of height, weight, and BMI values for healthy girls of the same age. Reference values for height, weight, and BMI of healthy, age-matched girls were sourced from the latest standards issued by the National Health Commission of the People’s Republic of China (11, 12).

### GnRH stimulation test

Venous blood (2 mL) was drawn from fasting female subjects at baseline (0 min). Gonadorelin was immediately injected subcutaneously in a dose of 2.5 μg/kg (up to a maximum dose of ≤100 μg), with 5 mL of normal saline added as an intravenous admixture. Further venous blood samples (collections of 2 mL each) were drawn at 30, 60, and 90 min post-injection, for a total of four collections. Serum was separated to quantify LH and FSH levels, with the peak values were used for assessment (2, 6).

### Color Doppler ultrasound examination

**Figure 1:**
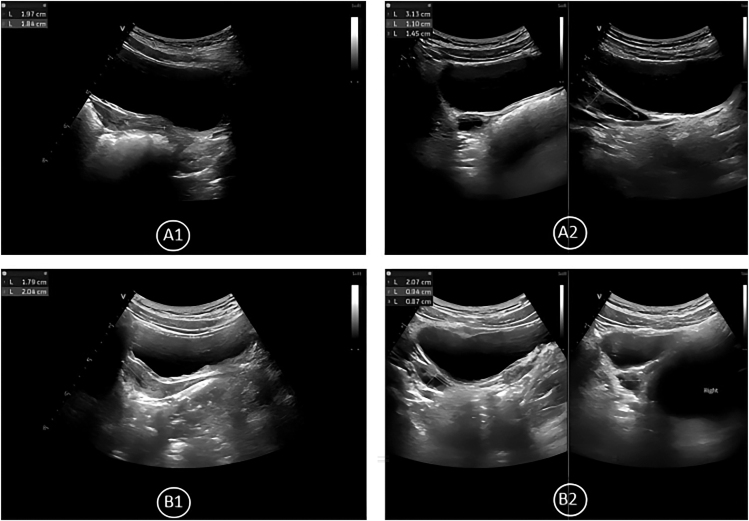
Radiographic BA, breast development, and uterine/ovarian imaging in the PT group. Representative case from the ICPP group (7 years old). (A) Pre-treatment: uterine volume, 1.11 mL; uterine body-to-cervix ratio, 1.6:1; endometrial lining, linear; right ovarian volume, 2.63 mL, containing five antral follicles >4 mm; largest antral follicle diameter, 1.0 cm. (B) Post-treatment: uterine volume, 0.84 mL; right ovarian volume, 0.89 mL, containing four antral follicles >4 mm; largest antral follicle diameter, 0.5 cm.

**Figure 2:**
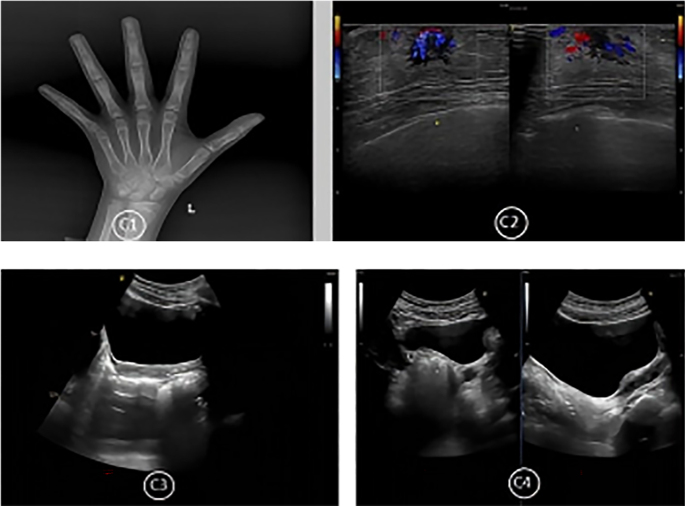
X-ray BA, breast development staging, and uterine/ovarian imaging for the PT cohort. Representative case from the PT group (6 years 11 months old). (C1) Left wrist, seven ossification centers visible; R series BA, 7 years 9 months; C series BA, 6 years 8 months (assessment, RUS-CHN method). (C2) Bilateral breast development, thickness of both glands 0.7 cm. (C3) Uterine volume, 0.91 mL; uterine body-to-cervix ratio, 1.1:1; endometrial lining, linear. (C4) Right ovarian volume, 1.45 mL, containing five antral follicles >4 mm; largest antral follicle diameter, 0.5 cm.

Participants were positioned, under parental supervision, in the supine position with full exposure of the chest. GE ViViD E95 and GE LOGIQ E9 systems, equipped with an L8-18i-D linear array probe, were used to measure bilateral breast thickness vertically behind the nipple. Two readings were taken and averaged. For the assessment of the uterus and ovaries, participants were instructed to maintain moderate bladder filling before the scan. The lower abdomen was exposed, and GE ViViD E95 and GE LOGIQ E9 systems, along with a C2-9-D convex array probe, were utilized to measure the following parameters in both the maximal longitudinal and transverse planes: uterus – anteroposterior (AP), transverse (TR), and longitudinal (LN) dimensions; ovaries – AP, TR, LN dimensions, number of follicles with a diameter >4 mm; and the maximum follicle diameter. Two readings were averaged for each measurement. Calculations: uterine volume (cm^3^) = AP × TR × LN × 0.523; average ovarian volume (cm^3^) = (right ovarian volume + left ovarian volume)/2; average number of follicles = (right ovarian follicles + left ovarian follicles)/2; average maximum follicle diameter = (right maximum diameter + left maximum diameter)/2. All dimensions were measured in centimeters (cm) and volumes in cubic centimeters (cm^3^). All operations were conducted by two doctors with over 3 years of experience in pediatric ultrasound diagnosis (Figs 1 and 2).

### Radiological examination

For X-ray BA assessment, all enrolled children underwent digital radiography of the posteroanterior wrist of the left hand. For radiographic positioning, the palm of the left hand faced downwards in full contact with the detector, the middle finger aligned perpendicularly with the longitudinal axis of the detector, fingers naturally spread apart, and the thumb at nearly 30° to the palm. The X-ray beam was centered vertically over the middle of the third metacarpal bone with a 90–110 cm source-to-image distance. A representative radiographic view is presented in Fig. 2. BA was interpreted with the Yitu BA Assessment Software using the RUS-CHN method (radius, ulna, short bones – China). The X-ray BAI was calculated as: BAI = (BA − CA)/CA. For pituitary MRI in ICPP group girls, the child wore headphones with the chin slightly tucked, shoulders pressed against the coil, head centered laterally and immobilized. A triangular cushion was used for fixation, with the positioning center at the nasion or glabella. For uncooperative children, parents accompanied them in the examination room. Image interpretation was performed by two radiologists with over 3 years of experience.

### Treatment protocol

The children in the ICPP treatment group were administered subcutaneously with Leuprorelin Acetate for Injection (Shanghai Livzon Pharmaceutical Co., Ltd, China) at an initial dose of 100 μg/kg. The medication was administered every 4 weeks, with the highest dose not exceeding 3.75 mg per administration. After 6 months of regular treatment, the entire experimental group of patients underwent a re-evaluation, which included measurements of height, weight, basal LH and FSH levels, an X-ray determination of BA, and an ultrasound examination. In addition, their growth velocity was calculated.

### Statistical analysis

Statistical analysis was conducted using SPSS version 27.0 and R Studio software. For quantitative data non-normally distributed variables were presented as medians with interquartile range (M (Q1, Q3)), while normally distributed variables were presented as means ± standard deviation (SD). For categorical data comparisons were made using the Chi-square test. For group comparisons (PT vs ICPP groups) an independent sample *t*-test was used for the comparison of normally distributed data. The Wilcoxon rank-sum test was used for comparing data that did not follow a normal distribution. In the development of a prediction model, the initial step involved screening clinically significant variables through univariate analysis. Variables from univariate analysis with a *P*-value < 0.1 were included in a multivariate LASSO-logistic regression analysis to identify independent predictor variables and construct a combined predictive model. The diagnostic ability was assessed using receiver operating characteristic (ROC) curves to compare the diagnostic efficacy of ultrasound parameters, the BAI, and their combination in the context of ICPP. Imaging parameters with a higher area under the curve (AUC) were selected for further assessment. For treatment efficacy analysis, within the ICPP group, pre- and post-treatment outcomes were compared using paired *t*-tests for normally distributed data and paired Mann–Whitney U tests for non-normally distributed data. Binary logistic regression was employed to create a combined evaluation metric, which was also utilized to compare single ultrasound and BAI parameters against therapeutic outcomes. A two-sided *P*-value of <0.05 was used as the significance level.

## Results

### Comparison of parameters between the CPP and PT groups

No statistically significant difference in age was observed between the two groups of girls (*P* > 0.05). The mean age of the ICPP group was 7.73 ± 0.78 years, which is consistent with findings from multiple studies: the Hainan, China, study (13) (216 ICPP girls) reported a mean onset age of 7.15 ± 1.06 years, and the Xi’an, China, study (20) (120 ICPP girls) found a mean onset age of 7.88 ± 1.76 years. Although existing studies included younger patients, none of them validated their conclusions using these patients as independent cohorts. Height, weight, and BMI were significantly higher in the ICPP group compared to the PT group: height ΔSDS in the ICPP group was 1.7 (> +1.5, suggesting accelerated growth, which may indicate precocious puberty), whereas in the PT group it was 0.61 (ranging from −0.1 to +1.5, indicating normal growth); weight SDS in the ICPP group was 3.44 (> +2.0, indicating overweight), whereas in the PT group it was 0.3 (ranging from −1.0 to +1.0, within the normal range); BMI SDS in the ICPP group was 5.48 (> +3.0, indicating severe obesity), whereas in the PT group it was 2.42 (> +2.0, indicating obesity). Basal LH, FSH, and LH/FSH ratios were significantly elevated in the ICPP group. According to the Chinese Expert Consensus on Diagnosis and Treatment of Central Precocious Puberty (2), a basal LH level >0.2 U/L may serve as a screening indicator for pubertal activation, but an LH level <0.2 U/L does not rule out CPP. Clinical correlation and GnRH stimulation tests are recommended when necessary. Therefore, definitive reference ranges for basal sex hormones in children remain uncertain due to multifactorial influences. Among 96 girls with CPP who underwent pituitary MRI, six cases of Rathke’s cysts were identified. The mean pituitary height was 0.52 ± 0.12 cm (comparable to that found by Zhang *et al.* (14), who found a mean pituitary height 0.57 ± 0.09 cm in 21 ICPP girls). The BAI and color Doppler ultrasound parameters in breast (mean bilateral breast thickness), uterus (longitudinal (LN), anteroposterior (AP) and transverse (TR) measurements; uterine volume; uterine body/cervix ratio; endometrial development), ovaries (mean bilateral ovarian longitudinal diameter; mean ovarian volume; mean number of follicles over 4 mm; mean largest follicle diameter) were all significantly higher in the ICPP group than in the PT group (*P* < 0.05). Diagnostic accuracy varied among imaging parameters: best accuracy (AUC >0.8) was found in mean breast thickness, uterine AP measurement, and uterine volume; intermediate accuracy (AUC >0.7) was found in uterine LN and TR measurements, ovarian volume, and BAI. Refer to Tables 1 and 2 for detailed information.

**Table 1:** General information, hormonal levels, bone age, and ultrasound findings.

Group	ICPP	PT	*t*/*Z*/*X*^2^	*P*-value
Age (y)	7.73 ± 0.78	7.54 ± 0.75	*t* = 1.738	0.084
Height (cm)				
Measured height	134.62 ± 6.94	128.77 ± 7.03		
Mean height of age-matched healthy girls	127.55 ± 4.96	127.15 ± 4.67		
Height SDS	1.4	0.34		
Target height (TH)	158.39 ± 2.92	158.59 ± 2.70		
TH-SDS	−0.3	−0.27		
ΔSDS	1.7	0.61		
Weight (kg)				
Measured weight	32.22 ± 6.15	26.58 ± 4.87	*t* = 6.994	<0.001
Mean weight of age-matched healthy girls	24.71 ± 2.18	25.53 ± 3.46		
Weight SDS	3.44	0.30		
BMI (kg/m^2^)	17.68 ± 2.49	15.93 ± 1.95	*t* = 5.375	<0.001
Mean BMI of age-matched healthy girls	15.16 ± 0.46	14.91 ± 0.42		
BMI-SDS	5.48	2.42		
Basal sex hormones				
Basal LH (IU/L)	1.60 (1.07, 2.62)	0.12 (0.08, 0.21)	*Z* = 11.130	<0.001
Basal FSH (IU/L)	4.74 ± 3.09	2.27 ± 1.28	*t* = 7.231	<0.001
LH/FSH ratio	0.41 (0.23, 0.85)	0.07 (0.05, 0.10)	*Z* = 10.576	<0.001
Imaging findings				
Bone age index (BAI)	0.23 ± 0.10	0.13 ± 0.12	*t* = 6.425	<0.001
Pituitary height (cm)	0.52 ± 0.12			
Ultrasound findings				
Mean bilateral breast thickness (cm)	0.95 ± 0.32	0.63 ± 0.21	*t* = 8.372	<0.001
Uterine longitudinal diameter (cm)	2.59 ± 0.62	2.10 ± 0.40	*t* = 6.536	<0.001
Uterine anteroposterior diameter (cm)	1.35 ± 0.50	0.90 ± 0.29	*t* = 7.706	<0.001
Uterine transverse diameter (cm)	2.30 ± 0.68	1.77 ± 0.34	*t* = 6.724	<0.001
Uterine body/cervix ratio	1.28 ± 0.27	1.17 ± 0.18	*t* = 3.044	0.003
Uterine volume (mL)	5.11 ± 4.97	1.84 ± 1.01	*t* = 6.303	<0.001
Mean bilateral ovarian longitudinal diameter (cm)	3.00 ± 0.45	2.74 ± 0.41	*t* = 4.092	<0.001
Average bilateral ovarian volume (mL)	3.31 ± 1.92	2.24 ± 0.93	*t* = 4.908	<0.001
Mean number of follicles >4 mm in bilateral ovaries (*n*)	5.64 ± 1.75	4.54 ± 1.66	*t* = 4.413	<0.001
Mean diameter of the largest follicle in the bilateral ovaries (cm)	0.63 ± 0.15	0.58 ± 0.21	*t* = 2.066	0.040
Endometrial development rate	Prepubertal 66.7% (*n* = 64)	Prepubertal 92.6% (*n* = 87)	*X*^2^ = 19.510	<0.001
	Pubertal 33.3% (*n* = 32)	Pubertal 7.4% (*n* = 7)		

**Table 2:** Comparison of the sensitivity and specificity of ultrasound parameters and bone age indices in diagnosing ICPP.

Variable	AUC	*P*-value	95% CI	Optimal threshold	Sensitivity	Specificity	Youden’s index
Mean bilateral breast thickness (cm)	0.80	<0.001	0.74–0.86	0.825	0.63	0.82	0.44
Uterine longitudinal diameter (cm)	0.75	<0.001	0.68–0.82	2.450	0.56	0.85	0.41
Uterine anteroposterior diameter (cm)	0.81	<0.001	0.75–0.87	1.050	0.69	0.78	0.47
Uterine transverse diameter (cm)	0.77	<0.001	0.70–0.84	1.950	0.72	0.70	0.42
Uterine body/cervix ratio	0.63	0.003	0.55–0.71	1.250	0.59	0.67	0.26
Uterine volume (mL)	0.82	<0.001	0.76–0.88	2.512	0.72	0.85	0.57
Mean bilateral ovarian longitudinal diameter (cm)	0.66	<0.001	0.58–0.73	2.775	0.66	0.73	0.34
Mean number of follicles >4 mm in bilateral ovaries (*n*)	0.68	<0.001	0.50–0.66	5.500	0.60	0.73	0.34
Mean diameter of the largest follicle in the bilateral ovaries (cm)	0.578	0.062	0.50–0.66	0.575	0.67	0.47	0.14
Average bilateral ovarian volume (mL)	0.72	<0.001	0.65–0.79	2.530	0.67	0.70	0.37
Bone age index (BAI)	0.76	<0.001	0.69–0.83	0.133	0.85	0.57	0.43

### Development of LASSO-logistic predictive model and nomogram

**Figure 3:**
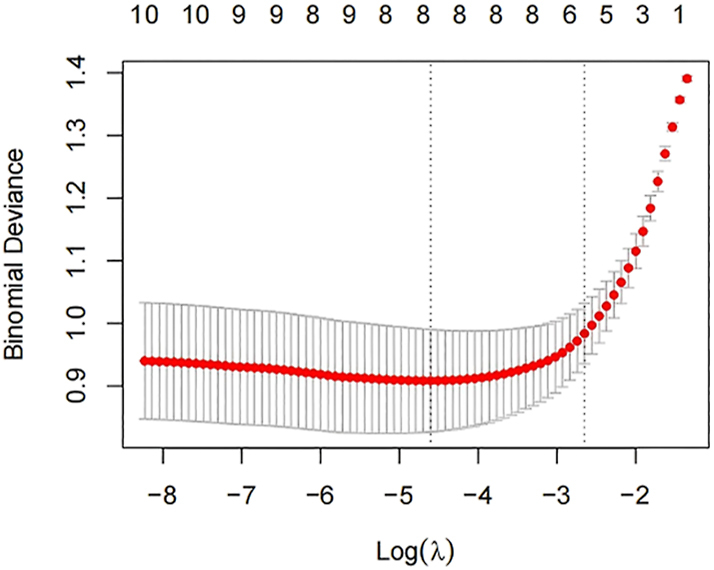
Lasso regression ten-fold cross-validation plot. X-axis, logarithm of λ (logλ). Y-axis, mean squared error (MSE). Dashed lines: lambda.min (optimal λ value corresponding to the minimum cross-validated MSE); lambda.1se (largest λ value within one standard error of the minimum MSE, selecting a simpler model while maintaining comparable predictive performance).

**Figure 4:**
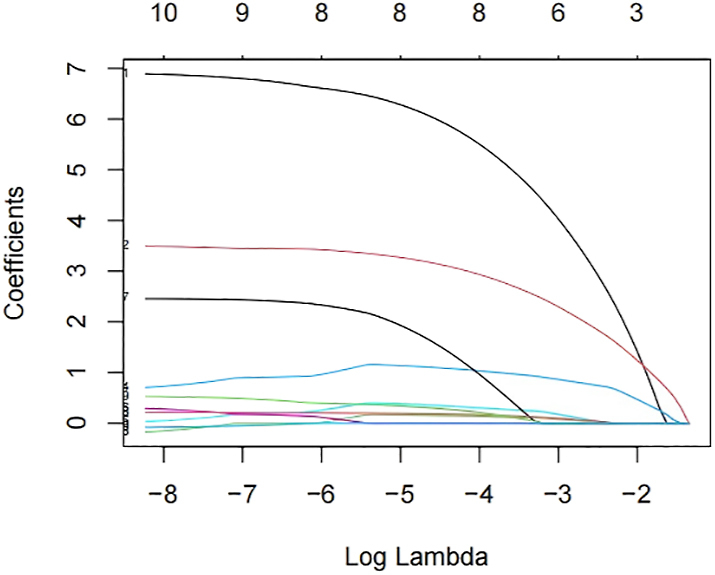
Lasso coefficient path plot. Under the minimum MSE criterion (λ.min), eight variables were selected, whereas the 1-SE criterion (λ.1se) identified a more parsimonious model with six variables.

**Figure 5:**
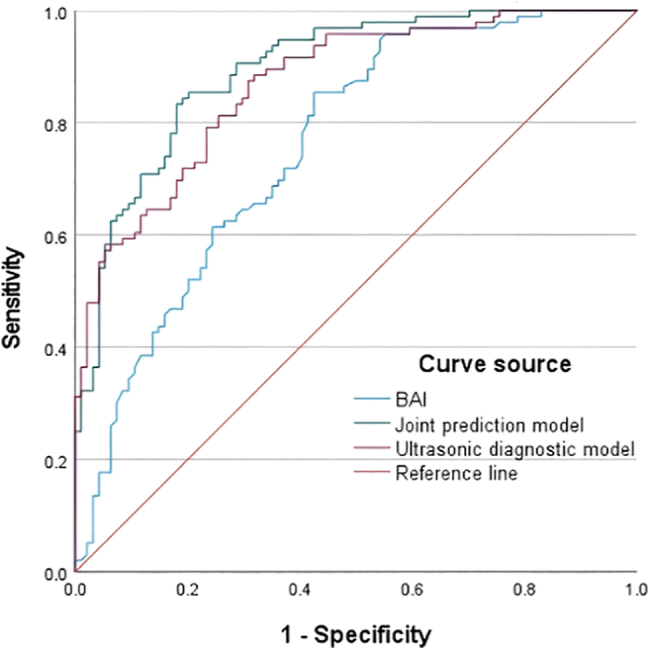
ROC curve of the predictive model.

**Figure 6:**
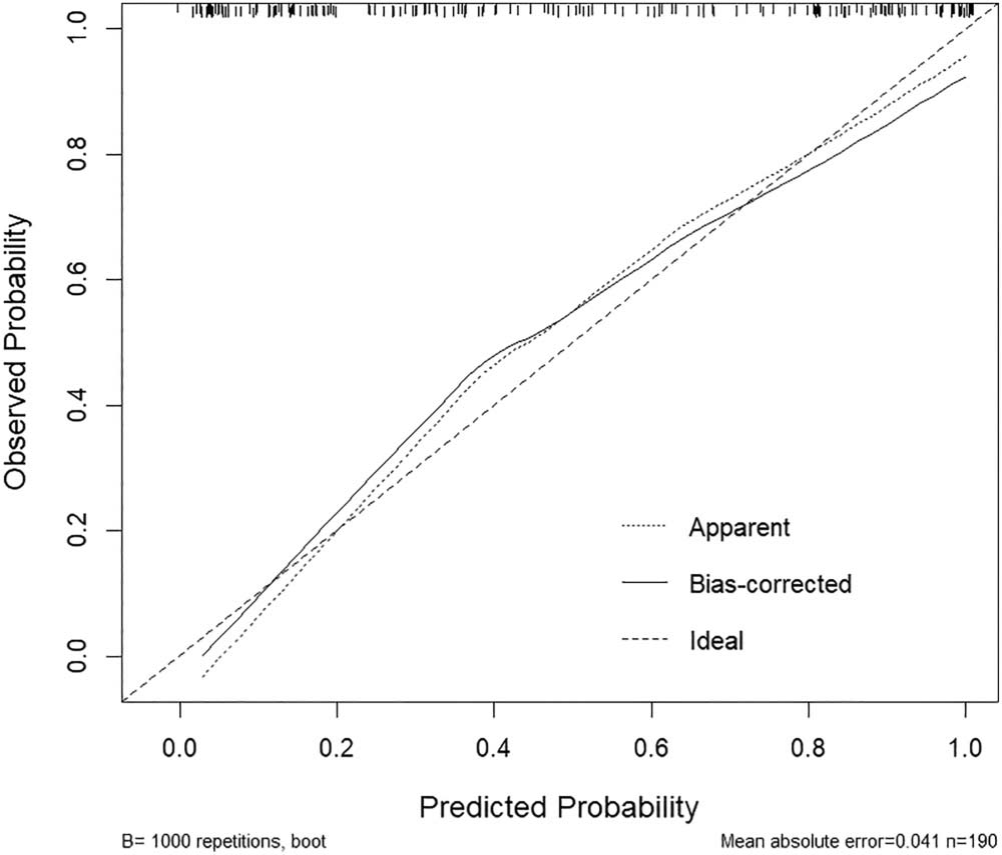
Calibration curve of the predictive model.

In a study of 190 females, LASSO regression was employed to screen predictor variables with the diagnosis of ICPP as the outcome variable (refer to Figs 3 and 4). The optimal lambda (λ) value was ascertained through ten-fold cross-validation. To ensure model stability, the minimum lambda (λ.min) was utilized to identify eight variables significantly associated with ICPP (BAI, mean breast thickness, uterine longitudinal dimension, uterine anteroposterior (AP) dimension, uterine transverse (TR) dimension, endometrial development, mean number of bilateral ovarian follicles >4 mm, and mean bilateral ovarian longitudinal diameter). These variables were then incorporated into a logistic regression model: logit (*P*) = −7.641 + 6.040 × BAI + 3.166 × mean breast thickness + 0.155 × uterine longitudinal dimension + 1.104 × uterine AP dimension + 0.361 × uterine TR dimension + 1.615 × endometrial development + 0.186 × mean number of ovarian follicles >4 mm + 0.303 × mean ovarian longitudinal diameter. The LASSO-logistic model demonstrated superior diagnostic accuracy with an AUC of 0.895 (95% CI: 0.851–0.938). When compared to individual models, the ultrasound-only model achieved an AUC of 0.869 (95% CI: 0.820–0.918), and the BAI-only model had an AUC of 0.758 (95% CI: 0.690–0.826). The Hosmer-Lemeshow goodness-of-fit test provided satisfactory calibration (*χ*^2^ = 10.158, *P* = 0.144). The model was strongly discriminating and well-calibrated, as evidenced by Figs 5 and 6.

**Figure 7:**
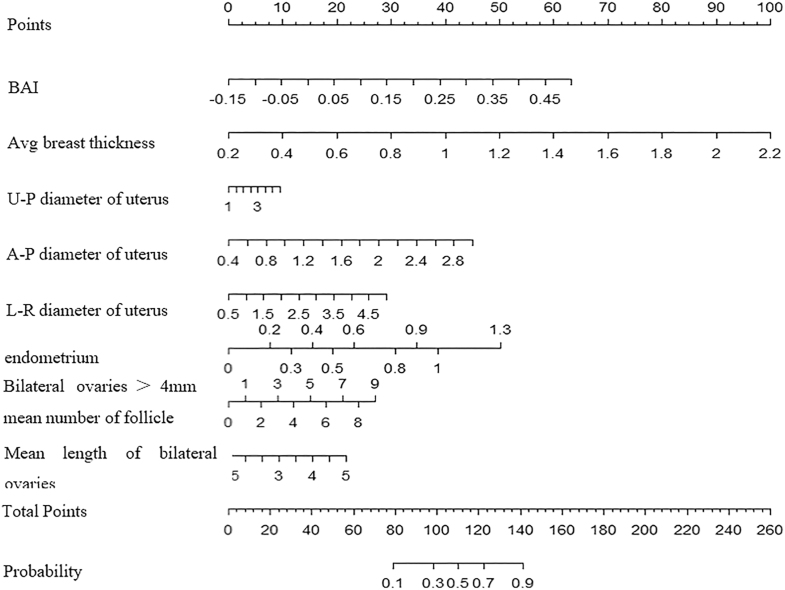
Nomogram for diagnosing ICPP based on predictive variables. Variables on the left (selected by LASSO regression): BAI, average breast thickness, uterine U-P diameter, uterine A-P diameter, uterine L-R diameter, endometrium, mean number of follicles >4 mm in bilateral ovaries, mean length of bilateral ovaries. The nomogram comprises of variable scales (where the numerical range next to each variable indicates its possible values) and line segment length (where the length of the line segment reflects the relative contribution of each variable to the model’s prediction of ICPP occurrence, longer segments = greater impact). The ‘Points’ row assigns a score to each variable based on its specific value; the ‘Total Points’ row sums all individual points to calculate the total points, which correlates with the predicted risk of ICPP; the ‘Probability’ row converts the total points into the estimated probability of an ICPP diagnosis.

Based on the results of the LASSO-logistic regression analysis, the following variables were utilized to construct a nomogram model for diagnosing ICPP: BAI, uterine longitudinal dimension, uterine anteroposterior (AP) dimension, uterine transverse (TR) dimension, endometrial development, the mean number of bilateral ovarian follicles larger than 4 mm, and the mean bilateral ovarian longitudinal diameter. The nomogram is depicted in Fig. 7.

### Comparison of pre- and post-treatment parameters in the ICPP group

Compared to pretreatment values, the following parameters were significantly reduced (*P* < 0.05) after treatment: LH, FSH, BAI, uterine longitudinal dimension, uterine anteroposterior (AP) dimension, uterine transverse (TR) dimension, uterine volume, uterine body-to-cervix ratio, mean bilateral ovarian longitudinal diameter, mean ovarian volume, mean number of ovarian follicles, and mean maximum follicle diameter. Among these parameters, those with greater diagnostic efficacy (AUC >0.7) for therapeutic monitoring were identified as: uterine AP dimension, mean bilateral ovarian longitudinal diameter, mean number of ovarian follicles ≥4 mm, mean maximum follicle diameter, and mean ovarian volume. Uterine longitudinal dimension and BAI demonstrated greater specificity. When integrated into a combined evaluation model, the parameters achieved an AUC of 0.877, with 75% sensitivity and 87% specificity, surpassing the outcomes of individual assessments based solely on ultrasound or BAI (refer to Tables 3 and 4). This combination serves as an effective supplement and optimization to assessments that rely on a single parameter.

**Table 3:** General information, hormonal levels, bone age, and ultrasound findings before and after treatment.

Group	Pre-treatment (mean ± SD)	Post-treatment (mean ± SD)	*Z*/*t*/*X*^2^	*P*-value
Height after 6 months of treatment	134.62 ± 6.94	139.01 ± 6.40		
Growth velocity		6.36 ± 3.05		
LH (IU/L)	2.10 ± 1.60	1.22 ± 0.86	*t* = 4.827	<0.001
FSH (IU/L)	4.74 ± 3.09	2.95 ± 2.11	*t* = 6.586	<0.001
LH/FSH	0.62 ± 0.54	0.65 ± 3.05	*t* = 0.208	0.836
Bone age index (BAI)	0.23 ± 0.10	0.17 ± 0.07	*t* = 7.728	<0.001
Ultrasound findings				
Uterine superior-inferior diameter (cm)	2.59 ± 0.62	2.33 ± 0.43	*t* = 4.937	<0.001
Anteroposterior diameter of uterus (cm)	1.35 ± 0.50	1.06 ± 0.38	*t* = 7.491	<0.001
Uterine left-right diameter (cm)	2.29 ± 0.68	2.03 ± 0.48	*t* = 4.879	<0.001
Uterine corpus-to-cervix ratio	1.28 ± 0.27	1.17 ± 0.16	*t* = 3.656	<0.001
Uterine volume (mL)	5.11 ± 4.97	2.94 ± 2.44	*t* = 5.985	<0.001
Mean length of bilateral ovaries (cm)	3.00 ± 0.45	2.60 ± 0.39	*t* = 7.900	<0.001
Mean volume of bilateral ovaries (mL)	3.31 ± 1.92	1.99 ± 1.20	*t* = 7.649	<0.001
Mean maximum follicle diameter in bilateral ovaries (cm)	0.62 ± 0.15	0.40 ± 0.24	*t* = 9.516	<0.001
Mean number of follicles >4 mm in both ovaries (*n*)	5.64 ± 1.75	3.24 ± 2.01	*t* = 10.263	<0.001

**Table 4:** ROC results of ultrasound and bone age index in diagnosing ICPP before and after treatment.

Variable	AUC	*P*-value	95% CI	Sensitivity	Specificity	Youden’s index
Uterine superior-inferior diameter (cm)	0.624	0.003	0.55–0.70	0.36	0.84	0.21
Antero-posterior uterine diameter (cm)	0.703	0.000	0.63–0.78	0.69	0.64	0.32
Uterine left-right diameter (cm)	0.616	0.005	0.54–0.70	0.72	0.46	0.18
Uterine body-to-cervix ratio	0.643	0.001	0.57–0.72	0.59	0.69	0.28
Uterine volume (mL)	0.679	0.000	0.70–0.76	0.72	0.62	0.34
Mean length of bilateral ovaries (cm)	0.747	0.000	0.68–0.82	0.66	0.73	0.35
Mean number of follicles >4 mm in both ovaries (*n*)	0.816	0.000	0.76–0.87	0.60	0.73	0.47
Mean maximum follicle diameter of bilateral ovaries (cm)	0.836	0.000	0.78–0.89	0.67	0.88	0.55
Mean volume of bilateral ovaries (mL)	0.796	0.000	0.73–0.86	0.67	0.70	0.51
Bone age index (BAI)	0.680	0.000	0.61–0.76	0.85	0.57	0.30
Combined assessment	0.877	0.000	0.83–0.93	0.75	0.87	0.6

## Discussion

The incidence of precocious puberty in China has been steadily increasing each year, closely related to unfavorable lifestyle habits (such as excessive screen time, poor sleep, a diet of high-calorie foods of poor quality, inactivity, and the consumption of bottled water, among others) (15, 16, 17, 18), as well as hereditary predispositions (18, 19). The treatment of the physical and psychological effects of precocious puberty involves a two-pronged approach: i) enhancing education to increase awareness among affected children and their parents about developmental differences from the general population, to correct lifestyle habits, and to foster a positive self-perception; ii) implementing early clinical interventions. According to previous studies (4, 20, 21), a linear relationship has been identified between the increase in BMI due to overweight/obesity and the onset of precocious puberty, complicating differential diagnosis. This finding is further supported by the outcomes of recent studies, which indicate that girls with CPP have a higher BMI compared to those with isolated PT.

The gold standard for diagnosing ICPP is the GnRH stimulation test; however, its limitations, including low sensitivity and the requirement for multiple blood draws, highlight the necessity for cost-effective, noninvasive imaging alternatives such as ultrasound and X-rays. Recent evidence increasingly supports the use of pelvic ultrasound as a valid diagnostic tool for ICPP. This study demonstrates that a uterine length >3.21 cm can serve as a specific diagnostic criterion for ICPP, with a specificity of 85%. These findings are consistent with China’s latest guidelines, which propose a uterine body length >3.2 cm as the diagnostic threshold for CPP, with a specificity of 82%. Most previous studies have identified uterine volume as the optimal diagnostic parameter for ICPP, though reported cutoff values vary significantly. Wen *et al.* (22) and Zarei *et al.* (23) documented uterine volume cutoffs of 1.09 and 1.40 mL, with sensitivities of 91.66 and 75.27%, and specificities of 77.60 and 75.27%, respectively, findings highly consistent with our study. Among single diagnostic indicators by ultrasound, uterine volume demonstrated the highest AUC (0.82), with a cutoff of 2.512 mL yielding 72% sensitivity and 85% specificity, second only to combined diagnostic approaches. This study further revealed that the uterine anteroposterior diameter and mean bilateral breast thickness also possess considerable diagnostic value, with AUCs of 0.81 and 0.80, cutoffs of 0.150 and 0.825, sensitivities of 69 and 63%, and specificities of 78 and 82%, respectively. Variability in the optimal parameters and corresponding thresholds may indicate racial differences, sample size variations, or differences in study design. BA, a biomarker of biological maturity, is further differentiated by the BAI, which measures the quantitative development of the individual’s skeletal system (24). This study constructed a LASSO-logistic regression prediction model that incorporates optimal imaging indicators. The model demonstrated high diagnostic value, with an AUC of 0.895, a sensitivity of 84%, and a specificity of 81%. Its diagnostic efficacy surpassed that of both the ultrasound-only model (AUC, 0.869; sensitivity, 88%; specificity, 69%) and the BAI model (AUC, 0.758; sensitivity, 85%; specificity, 57%). Note that uterine and ovarian volumes were excluded from variable selection due to multicollinearity with linear measures (e.g., uterine volume with AP/TR/LN dimensions). The LASSO algorithm enhanced model stability by addressing multicollinearity, making it an effective noninvasive diagnostic tool for ICPP, particularly in its early stages. Current guidelines (2, 25) recommend gonadotropin-releasing hormone agonist (GnRHa) therapy for girls with ICPP. GnRHa treatment effectively controls accelerated growth, reduces sex hormone levels, and improves final adult height. Post-treatment evaluation in our ICPP cohort revealed significant diagnostic parameters (AUC >0.7), including uterine anteroposterior diameter, mean bilateral ovarian longitudinal diameter, mean number of follicles >4 mm, mean maximum follicle diameter, and mean ovarian volume. The BAI showed an AUC of 0.68. This differential response may be attributed to GnRHa’s mechanism: by suppressing hypothalamic GnRH secretion, it reduces pituitary LH/FSH release, thereby directly inhibiting ovarian and uterine development (even causing regression). In contrast, the skeletal effects manifest more gradually. The combined assessment demonstrated superior efficacy (AUC, 0.877; sensitivity, 75%; specificity, 87%). Compared to isolated ultrasound or BA evaluation, our study indicates that combining ultrasound with the BAI provides complementary diagnostic information, enhances the discriminatory power for distinguishing ICPP from isolated PT, and enables effective monitoring of GnRHa therapeutic response, optimizing clinical decision-making. Therefore, the integration of ultrasound and radiographic BAI holds significant clinical value for both diagnosis and treatment surveillance in ICPP, meriting widespread clinical adoption (26). Notably, protracted peripheral precocity may trigger premature HPG axis activation, causing progression from peripheral to central precocity. This is evidenced by observed transitions from PT to ICPP in our longitudinal follow-up data.

This study has several limitations: the relatively small sample size and single-center data necessitate caution when generalizing the findings. Operator dependency in pelvic ultrasound measurements and subjectivity in BA assessments may introduce bias; further refinement is needed regarding standardized protocols and quality control. The evaluation of therapeutic value primarily focuses on diagnostic-treatment decision correlations, lacking long-term outcome tracking (e.g., final adult height) and assessment of personalized treatment adjustments based on the combined indicators. In addition, the established diagnostic model requires validation in independent external cohorts.

## Declaration of interest

The authors declare that there is no conflict of interest that could be perceived as prejudicing the impartiality of the work reported.

## Funding

North Sichuan Medical College Affiliated Hospital’s Talent Recruitment Project List (2022JB001) and Sichuan Provincial Natural Science Foundation Youth Project (2025ZNSFSC1751).

## Author contribution statement

Linli Kan contributed to the study design, data collection, data analysis, data interpretation, and article preparation. Deng He collected the clinical data and performed the analysis. Wensheng Yue contributed to the research design and manuscript revision.

## Ethical approval and consent to participate

Oral and written consent was obtained from all patients’ caregivers (parents or legal guardians) before enrollment in the study. This study followed the Declaration of Helsinki and was approved by the Medical Research Ethics Committee of Suining Central Hospital (KYLLKS20240204).
